# Multidimensional Recovery of Young Secondary Forests in Human‐Modified Tropical Landscapes

**DOI:** 10.1111/gcb.70874

**Published:** 2026-04-17

**Authors:** Tomonari Matsuo, Lourens Poorter, Lucy Amissah, Susan G. W. Laurance, Miguel Martínez‐Ramos, Jorge A. Meave, Frans Bongers, Masha T. van der Sande, Jazz Kok, Laura Marteijn, Luis Octavio Zavala, Iris Hordijk

**Affiliations:** ^1^ Forest Ecology and Forest Management Group Wageningen University & Research Wageningen the Netherlands; ^2^ Council for Scientific and Industrial Research‐Forestry Research Institute of Ghana Kumasi Ghana; ^3^ CSIR College of Science and Technology Accra Ghana; ^4^ Centre for Tropical Environmental and Sustainability Science (TESS) James Cook University Cairns Queensland Australia; ^5^ Instituto de Investigaciones en Ecosistemas y Sustentabilidad Universidad Nacional Autónoma de México Morelia Michoacán México; ^6^ Departamento de Ecología y Recursos Naturales, Facultad de Ciencias Universidad Nacional Autónoma de México Mexico City Mexico

**Keywords:** biodiversity, dry and wet forests, ecosystem multifunctionality, global climate and land‐use change, land‐use history, nature‐based solutions, pantropical analysis, secondary succession, tropical forests

## Abstract

Secondary succession is a widespread phenomenon in the Anthropocene due to global land‐use and climate change. Our ability to predict successional trajectories remains limited due to key knowledge gaps related to early secondary succession and how successional trajectories vary across socio‐ecological systems and multiple forest attributes. Therefore, we analyzed the first 5 years of secondary forest succession across six tropical landscapes (i.e., socio‐ecological systems) in three countries (Australia, Ghana, and Mexico) that differ in land‐use intensity and two main forest types (dry and wet). We established 122 permanent plots in recently abandoned agricultural fields, monitored them annually for up to 5 years, and quantified 12 forest attributes related to structure, diversity, functional composition, and biotic interactions. We found that a large variation in successional trajectories was explained by the six landscapes (average *r* ^2^ across 12 attributes is 54%; range: 18%–78%), indicating that succession is the result of a socio‐ecological system. An additional 39% of the variation (range: 19%–70%) was explained by plots occuring within landscapes, which reflects variation in landscape context and local land use intensity. Countries had a stronger impact on succession than forest type, indicating that the social component is more important early in succession, whereas the ecological component may become more important later in succession. Countries with lower land use intensity (e.g., subsistence agriculture, shorter duration of use, no mechanization) showed a higher start and speed of succession, as vegetation legacies can kickstart succession. Forest attributes followed distinct successional trajectories: forest structure and diversity increased over time, reflecting a deterministic component of succession, whereas functional composition and biotic interactions varied more with forest type, reflecting environmental filtering. These findings highlight the importance of integrating early succession, socio‐ecological systems, and multiple dimensions of forest recovery to better understand and predict forest succession in human‐modified tropical landscapes.

## Graphical abstract

Using up to 5 years of monitoring data following agricultural land abandonment across six tropical landscapes (three countries × two forest types), we analyzed how secondary tropical forests recover 12 attributes related to structure, diversity, functional composition, and biotic interactions. We found that: (1) successional trajectories varied widely across and within landscapes; (2) countries had a stronger influence on succession than forest type; and (3) forest attributes followed distinct trajectories. Together, these findings highlight the importance of integrating early successional dynamics, socio‐ecological context, and multiple dimensions of forest recovery to better understand and predict forest succession in human‐modified tropical landscapes.

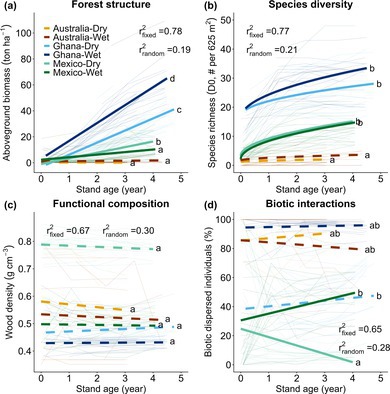

## 1 Introduction

Secondary succession is a widespread phenomenon in the Anthropocene, as human‐driven land‐use changes and natural disturbances clear vegetation, thus initiating the natural regeneration processes (Chazdon et al. 2025; Poorter et al. 2024). Although succession is typically described as the directional change in populations, communities, and ecosystems over time (Pickett et al. 1987; Poorter et al. 2023, 2024), successional pathways can vary widely both across and within landscapes (Meiners et al. 2015; Norden et al. 2015; Prach and Walker 2020; Walker and Chapin 1987). This spatial variability arises from differences in the start, speed, and direction of succession, which are strongly shaped during the first years of succession by environmental filtering, land‐use legacies, and priority effects (Cook‐Patton et al. 2020; Martin et al. 2013; Poorter, Rozendaal, et al. 2021). Such variability reduces the predictability of successional trajectories and thus limits the effective use of natural regeneration for ecosystem restoration. To better understand the drivers and mechanisms underlying spatial variability in successional trajectories, comprehensive cross‐site analyses of secondary succession are needed that (1) focus on the first 5 years of succession, when the start, speed, and direction of succession are defined, (2) compare successional pathways across landscapes that differ in land‐use history and forest types, and (3) incorporate multiple forest attributes simultaneously to capture the multidimensional nature of recovery. These knowledge gaps hinder our understanding of why succession advances rapidly in some landscapes but gets arrested or diverges in others (Norden et al. 2015). Here, we address these gaps by examining the first 5 years of secondary tropical forest succession, which constitutes a major share of human‐modified tropical landscapes and is central to global restoration efforts (Chazdon 2014; Chazdon et al. 2025).

Compared to primary succession, which occurs on previously unvegetated substrates, secondary succession typically progresses faster due to biological legacies from the previous vegetation such as developed soils, seed and seedling banks, resprouting stumps, and remnant trees (Egler 1954; Martínez‐Ramos et al. 2016; Yarranton and Morrison 1974). After two decades, secondary tropical forests attain, on average, 78% of neighboring old‐growth forest values across structural, taxonomic, functional, and soil attributes (Poorter, Craven, et al. 2021). Nevertheless, recovery rates vary widely both across and within landscapes, driven largely by spatial differences in climate (Cook‐Patton et al. 2020; Rozendaal et al. 2019, 2022), landscape forest cover (Grandjean et al. 2026; Pérez‐Cárdenas et al. 2021), and the type and intensity of land‐use practices (Hordijk, Poorter, Martínez‐Ramos, et al. 2024; Jakovac et al. 2021).

Dry and wet tropical forests typically differ in their successional pathways because contrasting climatic conditions shape regional species pools and the environmental filters that determine plant demographic rates (i.e., recruitment, growth, and survival) (Rozendaal et al. 2017). Generally, wetter forests attain faster recovery of structure and diversity during secondary succession because of larger regional species pools, more benign climatic conditions, and longer growing seasons (Poorter et al. 2016; Rozendaal et al. 2019). These forest types also diverge in functional composition and biotic interactions during succession (Lohbeck, Lebrija‐Trejos, et al. 2015; Poorter, Rozendaal, et al. 2021). In dry forests, hot and dry conditions at the beginning of succession impose strong environmental filters that favor drought‐tolerant species with high wood density, leaf mass per area, and nitrogen‐fixing ability (Gei et al. 2018; Lohbeck et al. 2013). In contrast, in wet forests, the highly weathered, nutrient‐poor soils may favor species that associate with arbuscular mycorrhizal fungi to enhance nutrient acquisition (Tedersoo et al. 2020). As a result, dry and wet tropical forests differ not only in the speed of succession for structure and diversity, but also in the direction of succession with respect to functional traits and biotic interactions.

The type and intensity of previous land use further modify these pathways. Low‐intensity, small‐scale shifting cultivation often retains seedling banks and remnant trees that kickstart succession with greater initial woody vegetation and accelerate early forest recovery through continuous recruitment and growth (Hordijk, Poorter, Martínez‐Ramos, et al. 2024; Titenwi et al. 2025). In contrast, intensive, large‐scale pastures tend to deplete soil nutrients, degrade soil seed banks, and introduce productive non‐native grass species that suppress tree regeneration, thereby decelerating or even arresting succession (Catterall 2016; Jakovac et al. 2021; Walker and Weston 1990). Studying succession across a wide range of socio‐ecological systems is therefore essential for identifying where natural regeneration is likely to occur, where it proceeds most rapidly, and where active interventions are needed to overcome barriers (Williams et al. 2024).

During succession, secondary forests develop differentially across multiple forest attributes related to structure, diversity, functional composition, and biotic interactions (Estrada‐Villegas et al. 2023; Guariguata and Ostertag 2001; Poorter, Rozendaal, et al. 2021). These attributes (i.e., different dimensions of recovery) are key to multiple ecosystem functions (e.g., forest productivity, habitat quality, and nutrient cycling) that underpin nature's contributions to people (e.g., climate change mitigation, biodiversity conservation, and soil health) (Hector and Bagchi 2007; Matsuo et al. 2026; Pinotti et al. 2012; Tedersoo et al. 2020; Teixeira et al. 2020). Therefore, a comprehensive understanding of multidimensional recovery (i.e., changes in multiple forest attributes) during the first 5 years of succession across socio‐ecological systems (e.g., forest types and land‐use history) is crucial both for advancing successional theory and for guiding the restoration of multifunctional tropical forests.

Here, we investigated how the start (initial values of forest attributes) and speed (rates of change in these attributes) of multidimensional forest recovery vary across six landscapes in three countries (Australia, Mexico, and Ghana) that differ in their agricultural land‐use histories, and across two forest types (dry vs. wet forests) (Table S1). We tested the following hypotheses: (1) large variation in successional trajectories occurs both across and within landscapes due to coarse‐ and fine‐scale differences in socio‐ecological conditions; (2a) the start of succession is greater in countries with lower land‐use intensity because biological legacies (e.g., resprouting individuals and seedlings) persist at the time of land abandonment; (2b) the speed of succession is faster in wet forests than in dry forests due to larger species pools, greater resource availability, and longer growing seasons; and (3) forest diversity and structure change more rapidly than functional composition and biotic interactions because of continuous species arrival and growth but slower species turnover.

## 2 Materials and Methods

### 2.1 Study System

We focused on secondary tropical forest succession following agricultural land abandonment, as pasture and shifting cultivation are the predominant drivers of tropical deforestation worldwide (Curtis et al. 2018), and the main areas where forest regrowth occurs (Chazdon et al. 2025; Williams et al. 2024). We focused on early succession right after land abandonment because (1) it is the phase when the fastest rates of successional change occur (Martin et al. 2013; Poorter, Craven, et al. 2021), and (2) it determines future successional pathways and therefore the extent to which forests can recover (Meiners et al. 2015; Rozendaal and Chazdon 2015; van Breugel et al. 2024). Most successional studies ignore this very early phase, and either start after several years when woody vegetation has already been established, or only measure larger trees (≥ 5 cm stem diameter) (e.g., Poorter, Craven, et al. 2021), thereby excluding regenerating saplings (≥ 1 cm diameter) that strongly contribute to initial structural and diversity recovery (van Breugel et al. 2006) and determine future canopy composition.

### 2.2 Study Sites

Research was conducted in three countries (Mexico, Ghana, and Australia), each on a different continent (North America, Africa, and Australia). This pantropical design enabled us to identify general patterns and context‐specific differences in succession. These three countries differ not only in their biogeographic conditions, with distinct floristic compositions, but also in their levels of socioeconomic development (Australia > Mexico > Ghana), which influence land tenure, management practices, land‐use intensity, and thus socio‐ecological systems (Table S1) (Baumann 2021; de Jong et al. 2025). Within each country, we selected two forest types (dry and wet) that represent the two main forest types in the lowland tropics, with potentially large differences in ecosystem functioning and succession (Rozendaal et al. 2019; van der Sande et al. 2024). An overview of all site locations and characteristics is presented in Table S1.

### 2.3 Plot Establishment and Vegetation Measurements

A total of 122 permanent plots (25 × 25 m) were established in recently abandoned pastures or agricultural land. Most plots had been completely cleared prior to agricultural activities, although remnant trees were sometimes retained for shade, fruit, or cultural reasons, or left standing due to constraints such as limited mechanization (e.g., in Ghana) (Matsuo et al. 2023). Several plots in Mexico were fenced where necessary to exclude cattle and prevent browsing. In most plots (78% of all plots), monitoring began immediately following land abandonment. A smaller number of plots were established 1 year (16%) or 2 years (6%) after abandonment due to temporary COVID‐related fieldwork restrictions.

All trees and shrubs with a stem diameter ≥ 1 cm at 30 cm above ground (Mexican dry forest) or at breast height (DBH, 1.3 m, all other sites) were identified and measured annually for up to 5 years, resulting in 478 plot‐census combinations. The lower measurement height in the Mexican dry forest was used because shrub species, which are common in this site, frequently branch at very low heights, making standard DBH measurements less practical, especially in very early stages of secondary succession. The 1 cm threshold ensured inclusion of small individuals critical for determining future forest composition.

### 2.4 Measurements of 12 Forest Attributes

We quantified 12 forest attributes from four complementary groups: (1) forest structure, (2) species diversity and late‐successional species, (3) functional composition, and (4) biotic interactions. To provide a coherent and balanced overview, we used three attributes per group. All attributes were derived from standardized measurements of aboveground woody vegetation.

#### 2.4.1 Forest Structure

We described forest structure using aboveground biomass, the size of the largest trees, and heterogeneity in forest structure.

##### 2.4.1.1 Aboveground Biomass (AGB)

AGB (in ton ha^−1^) was estimated using the pantropical tree allometric equations, using stem diameter, species‐specific stem wood density (WD, in g cm^−3^), and a measure of environmental stress (*E*) (Chave et al. 2014). When WD was unavailable for some species, we used the highest taxonomic resolution available (genus or family level) or the site‐average WD. *E* is a composite variable derived from climatic factors such as temperature seasonality, precipitation, and climatic water deficit, and is used to adjust the tapering factor to estimate tree height from DBH under different environmental conditions (Chave et al. 2014). Plot‐level AGB was calculated by summing individual tree biomass and scaling to a per‐hectare basis.

##### 2.4.1.2 Maximum Size (Size)

Maximum tree size was quantified as the stem diameter or height of the third‐largest trees in a plot. Large trees are of high conservation value because they provide habitat, shelter, and food for epiphytes, lianas, fungi, animals, and insects, and they contribute substantially to carbon sequestration and storage (Bordin et al. 2021; Lutz et al. 2021). Because maximum size can be strongly influenced by the presence of large remnant trees, we conservatively estimated it as the median of the five thickest or tallest individuals per plot. For plots with fewer than five individuals, we added hypothetical individuals with a diameter or height of zero. We used diameter‐based maximum size for the calculation of forest multifunctionality (*N* = 478), as these data were more complete, and height‐based maximum size for describing successional patterns (*N* = 441), as it better reflects forest structural development.

##### 2.4.1.3 Structural Heterogeneity

Structural complexity (dimensionless) captures the variation in tree sizes within a plot, reflecting vertical layering and forest maturity. We quantified structural complexity using the Gini coefficient of the stem basal area of all individual trees in a plot using the “dineq” R package (Schulenberg 2018). The Gini coefficient ranges from 0 (uniform stem basal area) to 1 (maximum variation). It was computed as the sum of all absolute differences in stem basal area across all pairwise combinations of the *N* individuals in the plot, divided by 2 × *N* ^2^ × mean stem basal area of all individuals, where *N* is the total number of individuals in the plot.

#### 2.4.2 Species Diversity and Composition

We described species diversity based on two measures derived from Hill numbers, and species composition based on the percentage of late‐successional species.

##### 2.4.2.1 Species Diversity

Species diversity was quantified using Hill numbers (Chao et al. 2014), which express diversity in units of “effective number of species,” providing a unified framework for comparing species richness and evenness. We calculated Hill numbers of order *q* = 0 and *q* = 1. The Hill number of order *q* = 0 (^0^ *D*) represents species richness, giving equal weight to all species, whereas the Hill number of order *q* = 1 [^1^ *D* = exp. (*H*′)] represents the number of equally abundant species implied by the Shannon diversity index, giving proportional weight to species abundances. Because plot size and sampling protocols were standardized across sites, diversity was calculated directly from the observed assemblages without rarefaction or sample‐coverage standardization.

##### 2.4.2.2 Late‐Successional Species

The percentage of late‐successional species was calculated for each plot and census year as an indicator of its similarity to old‐growth forests. For each country, the species' successional guild was derived from published studies, unpublished long‐term monitoring data, and expert knowledge (Hawthorne 1995; Hordijk, Poorter, Meave, et al. 2024; Kanowski et al. 2010; Lebrija‐Trejos, Meave, et al. 2010; Lebrija‐Trejos, Pérez‐García, et al. 2010; Matsuo, Bongers, et al. 2024; van Breugel et al. 2007). Species were classified as early‐, mid‐, or late‐successional, based on temporal dynamics, persistence during succession, and light requirements for growth and survival. Early‐successional species are predominantly light‐demanding, recruiting within the first 5 years of succession (< 5 years) (Peña‐Claros 2003; Uhl 1987) and dominating for the first 20 years (Finegan 1996). Mid‐successional species establish and grow under the canopy of early‐successional species, typically dominating succession between 20 and 100 years (Budowski 1965; Finegan 1996). Late‐successional species are shade‐tolerant, recruit continuously throughout succession, and dominate the canopy after 100 years until major disturbances reset succession (Finegan 1996; Swaine and Hall 1983). We assigned numerical values to species (early = 0, mid = 0.5, late = 1.0) and weighted them by their relative abundances in each plot. We weighted it by the relative abundance rather than basal area since basal area is strongly influenced by remnant trees, especially in the first years of succession. Species lacking successional guild information were excluded from the calculation, resulting in 83.3% coverage of plot–census combinations. Values were expressed as percentages, with higher values indicating a greater proportion of.

#### 2.4.3 Functional Composition

Functional trait composition of the community was described using the community‐weighted mean (CWM) trait values of stem wood density, leaf mass per area, and resprouting (see below for a description of the traits). CWMs represent the average trait value in a community weighted by species abundances and are closely linked to community assembly, ecosystem processes, and functioning (Lohbeck, Poorter, et al. 2015; Matsuo et al. 2025; van der Sande et al. 2017). CWM trait values were calculated by multiplying each species' trait value by its relative abundances within the plot and then summing across all species. Species without trait data were excluded from the calculation of CWM. Trait data were obtained locally at each study site to capture site‐specific environmental conditions (Kanowski et al. 2010; Lebrija‐Trejos, Pérez‐García, et al. 2010; Lohbeck, Poorter, et al. 2015; Matsuo et al. 2025; Matsuo, van der Sande, et al. 2024; Ortiz et al. 2023; Potts et al. 2022; Romero et al. 2020; Sandoval‐Granillo and Meave 2023) and were supplemented with information from global databases when local measurements were unavailable (Chave et al. 2006; Falster et al. 2021; Zanne et al. 2009). Because trait data were collected at the species level, plastic responses to successional stage could not be incorporated, meaning that successional changes in CWM reflect only species turnover. To ensure robust estimates, we restricted analyses to plots for which trait values were available for more than 80% of individuals, resulting in an average of 82.4% plot‐census combinations retained (range: 79.1%–84.9%).

##### 2.4.3.1 Wood Density (WD)

Stem WD (in g cm^−3^) is the wood dry mass divided by the wood fresh volume. WD is important for carbon cycling because low WD increases stem hydraulic conductivity, photosynthetic carbon gain, and volumetric growth capacity, but decreases tissue density (Chave et al. 2009; Poorter et al. 2010), whereas high WD is associated with longer‐lived tissues and increased carbon residence time.

##### 2.4.3.2 Leaf Mass Per Area (LMA)

LMA (in g m^−2^) is the ratio of leaf mass to leaf area and reflects the cost of leaf construction. LMA is a key component of the leaf economics spectrum (Wright et al. 2004) and scales positively with leaf longevity but negatively with photosynthetic capacity (Onoda et al. 2011; Wright et al. 2004). Hence, it is related to forest productivity (Finegan et al. 2015).

##### 2.4.3.3 Resprouting

Resprouting is the ability to regenerate shoots or stems from existing structures (e.g., stumps, roots, or branches) following disturbance or damage (Pausas et al. 2016; Pausas and Keeley 2014). This strategy is particularly common in frequently disturbed or dry environments (Caplat and Anand 2009; Jakovac et al. 2016; Mesquita et al. 2015), enabling rapid recovery after major disturbances or stem dieback due to drought. In the field, each individual was visually assessed as originating from a seed or resprouting based on stem morphology and point of origin. Individuals connected to a visible stump or root collar were classified as resprouts, whereas those emerging independently from the soil were classified as seedlings. The percentage of resprouting individuals was then calculated per plot.

#### 2.4.4 Biotic Interactions

Biotic interactions were quantified as the percentage of trees belonging to species dispersed by biotic agents, capable of nitrogen fixation, or potentially forming arbuscular mycorrhizal associations. To ensure robust estimates, we restricted analyses to plots for which information was available for more than 80% of individuals, resulting in an average of 83.8% plot‐census combinations retained (range: 79.1%–86.2%).

##### 2.4.4.1 Biotic Seed Dispersal

Dispersal mode refers to the vector by which seeds are transported from the parent plant to new locations, playing a critical role in the recovery of species diversity and composition in naturally regenerating forests (Arroyo‐Rodríguez et al. 2017; Bello et al. 2024; Estrada‐Villegas et al. 2023). Information on the dispersal mode (e.g., birds, bats, mammals, wind, water, and gravity) for each species was collected from literature, books, and expert knowledge (Hordijk, Poorter, Meave, et al. 2024; Kanowski et al. 2010; Paetzold et al. 2018). Species were subsequently classified as either biotically dispersed (coded as 1) or abiotically dispersed (coded as 0).

##### 2.4.4.2 Nitrogen Fixation

Biological nitrogen (N) fixation is the result of a symbiotic relationship between certain tree species and *Rhizobium* bacteria. It provides the major nitrogen input to tropical forests and is an important source of nitrogen recovery during secondary tropical forest succession on abandoned agricultural fields (Batterman et al. 2013; Gei et al. 2018). We extracted information on whether tree species fix nitrogen or not from available databases (Tedersoo et al. 2018). Because actual N fixation rates depend on *Rhizobium* activity, which can be affected by local conditions, we assessed potential rather than realized fixation. Species were classified as nitrogen‐fixing (1) or non‐nitrogen‐fixing (0).

##### 2.4.4.3 Arbuscular Mycorrhizal Association

Arbuscular mycorrhizal (AM) associations are symbiotic relationships between *Glomeromycota* fungi and plant roots, facilitating the exchange of nutrients, especially phosphorus, in exchange for carbon from the plant. This mutualistic interaction is crucial for water and nutrient uptake in tropical forests, especially for immobile nutrients such as phosphorus. AM plays, therefore, an important role in nutrient cycling. We extracted information on whether tree species form AM associations from available databases (Soudzilovskaia et al. 2020; Yamawo and Ohno 2024). As with N fixation, we only considered species‐level potential, not realized mycorrhizal colonization. Species were classified as arbuscular mycorrhizal (1) or non‐arbuscular mycorrhizal (0).

### 2.5 Calculation of Forest Multifunctionality

Forest multifunctionality was calculated based on 12 forest attributes to assess the multidimensional recovery of multiple forest attributes during secondary succession. Each attribute was normalized to a 0–1 scale by subtracting its minimum value and dividing by its observed range to allow comparability across attributes with different units. We then calculated multifunctionality indices using the Hill‐number framework of order *q* = 1 (Chao et al. 2024), which weights attributes according to their relative contribution to overall forest multifunctionality. This framework integrates both the magnitude and evenness of multiple attributes while correcting for correlations among them to avoid redundancy. It interprets multifunctionality as the effective number of independent functions, and therefore increases when more attributes reach high performance values and when their contributions are more evenly distributed. Because the index represents the effective number of functions, its theoretical maximum equals the total number of attributes (i.e., 12). The Hill‐based multifunctionality index was computed for each plot and census year and used as a composite measure of multidimensional forest recovery. To ensure robust estimates, we restricted analyses to plots with trait and symbiotic information available for more than 80% of the individuals, resulting in 366 plot–census combinations (76.6% of the total dataset).

### 2.6 Statistical Analysis

We quantified tropical forest recovery by evaluating the start (initial values of forest attributes) and speed (annual rates of change in these attributes) of successional changes in 12 forest attributes related to structure, diversity, functional composition, and biotic interactions, as well as overall forest multifunctionality. For multifunctionality and all individual attributes, we fitted linear mixed‐effects models (LMMs) using the lmer function in the “lme4” package (Bates et al. 2015), using all 478 plot–census combinations (mean *N* = 423; range = 366–478). Fixed effects included country (Ghana, Mexico, Australia), forest type (dry, wet), stand age, and their three‐way interactions, and plot was included as a random intercept and slope to account for repeated measurements across censuses. In this framework, the model intercept represents the post‐abandonment value of each attribute (i.e., the starting condition at stand age = 0), and the slope represents its annual rate of change (i.e., the speed of succession) during the first 5 years. Estimated marginal means were extracted for both intercepts and slopes to compare differences among the six landscapes, three countries, and two forest types, with post hoc pairwise comparisons adjusted using the Sidak correction. Statistical significance was assessed at *α* = 0.05, and 95% confidence intervals were calculated for all model intercept and slope estimates.

To quantify variation in successional trajectories across and within sites, we partitioned the variance explained by fixed and random effects. Because country and forest type were included as fixed effects, the variance explained by the fixed effects reflects differences among sites/landscapes (i.e., among country × forest‐type combinations). In contrast, plot was included as a random effect to account for repeated measurements within each site; variance attributed to the random effects therefore represents within‐site variation (i.e., differences among plots within the same site). Such variation may arise from finer‐scale factors such as landscape context or soil conditions, although these variables were not explicitly included in the models.

To visualize how the 12 forest attributes are associated and how they contribute to forest multifunctionality during the first years of secondary succession, we used network analysis. The network was constructed based on pairwise Pearson correlations among the 12 forest attributes. To assess uncertainty, we estimated 95% confidence intervals for each pairwise correlation (*r*) using a resampling approach: 80% of the dataset was randomly selected 10,000 times, and pairwise correlations and network metrics were recalculated for each iteration (Poorter, Craven, et al. 2021).

## 3 Results

### 3.1 Multidimensional Tropical Forest Recovery During the First 5 Years of Succession

**FIGURE 1:**
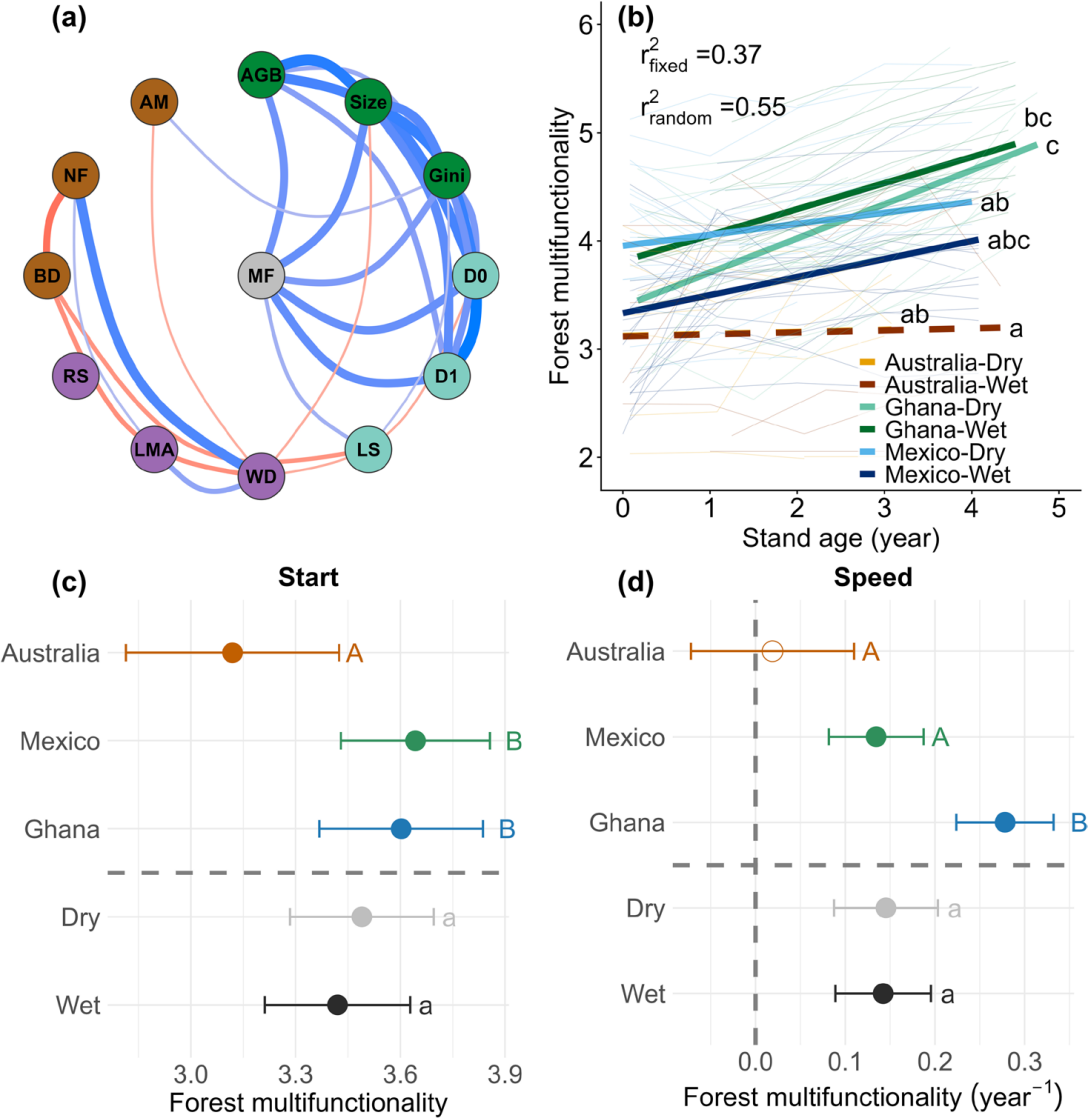
Multidimensional tropical forest recovery during the first 5 years of succession. (a) Correlation network among 12 forest attributes and overall multifunctionality, based on all plot‐census combinations that met the calculation criteria of functional composition and biotic interactions (*N* = 366). Multifunctionality was calculated across the 12 forest attributes and could theoretically range from 0 to 12. Line color and thickness indicate the direction and strength of correlations (blue = positive, red = negative); darker and thicker lines represent stronger relationships. Lines are shown only for significant pairwise correlations (i.e., with a 95% confidence interval that does not overlap with zero) that exceed a correlation threshold (|*r*| > 0.3), highlighting moderate to strong relationships (see Figure S1a for all significant correlations). (b) Successional increases in forest multifunctionality across six sites/landscapes (country‐forest type combinations). A linear mixed‐effects model was fitted with multifunctionality as the response variable, country (Ghana, Mexico, Australia), forest type (dry, wet), stand age, and their three‐way interactions as fixed effects, whereas plot was included as a random intercept and slope to account for repeated measurements across censuses. Regression lines from linear mixed models are shown for each site, with different letters indicating significant differences in slopes based on post hoc Sidak tests. Coefficients of determination (*r* ^2^) are shown for fixed (site) and random (i.e., repeated measurements per plot) effects. (c, d) Estimated marginal means for the (c) start (initial value; intercept) and (d) speed (annual rate of change; slope) of multifunctionality across countries and between forest types. Capital letters denote significant post hoc differences among countries, and lowercase letters denote differences between dry and wet forests. Closed circles indicate significant (c) intercepts or (d) slopes, whereas open circles indicate non‐significant values. Attribute abbreviations: Aboveground biomass (AGB; ton ha^−1^), diameter of the third thickest individual (size; cm), Gini coefficient of tree basal area within each plot (Gini), species richness based on Hill number of order 0 (*D*_0_; per 625 m^2^), exponentiated Shannon diversity based on Hill number of order 1 (*D*_1_; per 625 m^2^), percentage of later‐succession species (LS; %), community‐weighted mean wood density (WD; g cm^−3^), community‐weighted mean leaf mass per area (LMA; g m^−2^), percentage of resprouting individuals (RS; %), percentage of biotically dispersed trees (BD; %), percentage of nitrogen‐fixing trees (NF; %), and percentage of trees with the capacity to form arbuscular mycorrhizal association (AM; %). For a detailed description of the attributes, see Section 2.

Multifunctionality during the first 5 years was more strongly correlated with structural and diversity attributes than functional composition and biotic interactions (Figure 1a, Figure S1a,b). This pattern likely reflects greater variation across space in structure and diversity and greater variation in their recovery over time, making these dimensions more detectable in the integrated multifunctionality signal. Forest multifunctionality increased significantly in both dry and wet forests in Mexico and Ghana, but not in Australia (Figure 1b). Both the starting values and the speed of multidimensional recovery (intercepts and rates of changes in multifunctionality) differed significantly among countries but not between dry and wet forests (Figure 1c,d). Starting values were higher in Ghana and Mexico than in Australia, and the speed of succession was faster in Ghana than in Mexico and Australia.

### 3.2 Countries Differ Largely in the Start and Successional Speed of 12 Forest Attributes

**FIGURE 2:**
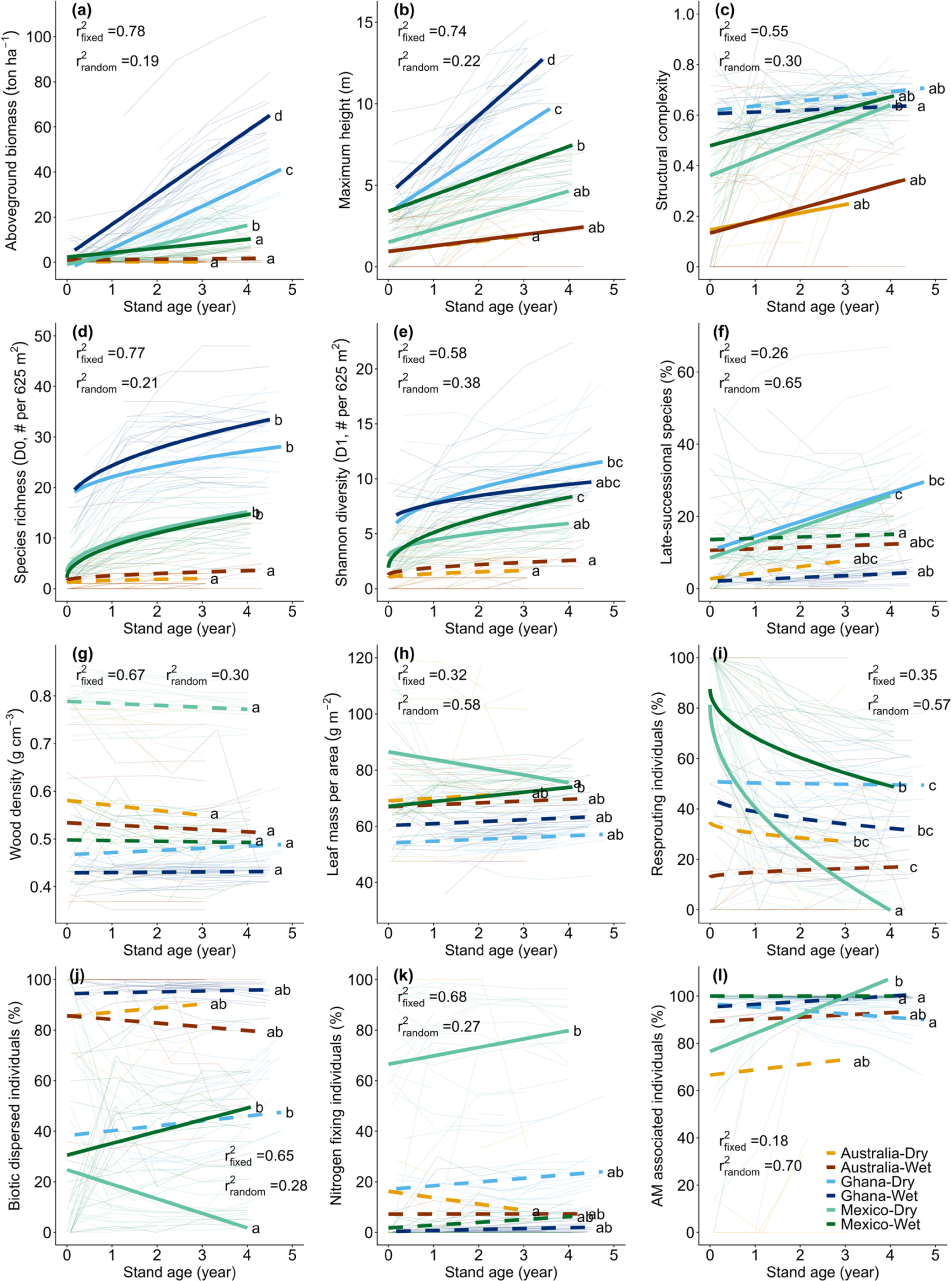
Successional changes in 12 forest attributes across six study landscapes. Thin lines represent repeated measurements for each plot, and thick lines show successional trends estimated using linear mixed‐effects models for each site/landscape (six country‐forest type combinations). Continuous thick lines indicate significant trends, whereas dashed thick lines indicate non‐significant trends. Letters along the lines denote post hoc comparisons of slopes among landscapes; different letters indicate significantly different slopes. Coefficients of determination (*r* ^2^) are shown for fixed (site) and random (i.e., repeated measurements per plot) effects. Maximum height was estimated as the height of the third‐tallest individual. Structural complexity was calculated as the Gini coefficient of tree basal area within each plot for each census. Attribute abbreviation: Arbuscular mycorrhizal fungi (AM). For a detailed description of the attributes, see Section 2.

Countries differed significantly in the starting values of 10 attributes and in the speed of eight attributes (Figure 2, Table 1). Starting values of structural and diversity attributes (maximum height, structural heterogeneity, species richness, and Shannon diversity) were highest in Ghana, intermediate in Mexico, and lowest in Australia (Table 1). In contrast, starting values of functional composition and biotic interactions showed no consistent cross‐country pattern: Mexico had the highest community wood density and the greatest percentages of resprouting individuals and potential nitrogen fixers; Australia had the highest percentage of biotically dispersed species; and Ghana had the highest percentage of arbuscular mycorrhiza‐associated species but the lowest community leaf mass per area (Table 1).

**TABLE 1:** Start and speed of tropical forest succession across 12 forest attributes, countries, and forest types.

Attribute	Start of succession					Speed of succession				
	Australia	Mexico	Ghana	Dry	Wet	Australia	Mexico	Ghana	Dry	Wet
AGB	0.61 (A)	0.67 (A)	−0.08 (A)	−1.3 (a)	**2.1 (b)**	0.081 (A)	**3.1 (B)**	**11.6 (C)**	**4.5 (a)**	**5.3 (a)**
Height	**0.94 (A)**	**2.5 (B)**	**3.9 (C)**	**1.9 (a)**	**2.9 (b)**	**0.33 (A)**	**0.89 (B)**	**2.1 (C)**	**0.96 (a)**	**1.3 (b)**
Gini	**0.14 (A)**	**0.42 (B)**	**0.61 (C)**	**0.37 (a)**	**0.41 (a)**	**0.041 (AB)**	**0.059 (B)**	0.013 (A)	**0.041 (a)**	**0.035 (a)**
*D*_0_	1.4 (A)	**2.7 (A)**	**16.6 (B)**	**7.1 (a)**	**6.7 (a)**	0.26 (A)	**2.2 (B)**	**2.3 (B)**	**1.4 (a)**	**1.8 (a)**
*D*_1_	**1.1 (A)**	**2.4 (A)**	**5.3 (B)**	**2.9 (a)**	**3.0 (a)**	0.18 (A)	**0.83 (B)**	**0.87 (B)**	**0.59 (a)**	**0.66 (a)**
LS	**6.7 (A)**	**11.0 (A)**	**6.3 (A)**	**7.3 (a)**	**8.7 (a)**	1.1 (A)	**2.3 (A)**	**2.3 (A)**	**3.3 (b)**	0.45 (a)
WD	**0.56 (B)**	**0.64 (C)**	**0.45 (A)**	**0.61 (b)**	**0.49 (a)**	−0.0076 (A)	−0.0028 (A)	0.0025 (A)	−0.0034 (a)	−0.0018 (a)
LMA	**68.0 (B)**	**76.8 (B)**	**57.1 (A)**	**69.9 (a)**	**64.5 (a)**	0.80 (A)	−0.53 (A)	0.67 (A)	−0.38 (a)	1.0 (a)
RS	**23.8 (A)**	**84.4 (C)**	**48.3 (B)**	**55.7 (a)**	**48.7 (a)**	−0.41 (B)	**−10.3 (A)**	−1.3 (B)	**−5.3 (a)**	**−2.7 (b)**
BD	**85.6 (C)**	**27.7 (A)**	**66.3 (B)**	**49.5 (a)**	**70.2 (b)**	0.077 (A)	−0.55 (A)	1.1 (A)	−0.73 (a)	1.2 (a)
NF	**11.7 (A)**	**34.1 (B)**	**8.6 (A)**	**33.2 (b)**	3.1 (a)	−1.2 (A)	**2.2 (B)**	0.920 (AB)	0.76 (a)	0.51 (a)
AM	**77.9 (A)**	**88.3 (AB)**	**96.1 (B)**	**80.0 (a)**	**94.9 (b)**	1.6 (AB)	**3.9 (B)**	−0.13 (A)	**2.8 (a)**	0.70 (a)

*Note:* Estimated marginal means are shown for the start (initial value; intercept) and speed (annual rate of change; slope) of succession, derived from linear mixed‐effects models. Post hoc differences among countries (Australia, Mexico, Ghana) and between forest types (dry, wet) were tested using Sidak‐adjusted comparisons; different letters indicate significant differences at *p* < 0.05. Bold values denote significant model estimates (*p* < 0.05). Attribute abbreviations: Aboveground biomass (AGB; ton ha^−1^), height of the third tallest individual (Height; m), Gini coefficient of tree basal area within each plot (Gini), species richness based on Hill number of order 0 (*D*_0_; per 625 m^2^), exponentiated Shannon diversity based on Hill number of order 1 (*D*_1_; per 625 m^2^), percentage of late‐succession species (LS; %), community‐weighted mean wood density (WD; g cm^−3^), community‐weighted mean leaf mass per area (LMA; g m^−2^), percentage of resprouting individuals (RS; %), percentage of biotically dispersed trees (BD; %), percentage of nitrogen‐fixing trees (NF; %), and percentage of trees with the capacity to form arbuscular mycorrhizal association (AM; %). For a detailed description of the attributes, see Section 2. Estimated marginal means with 95% confidence intervals are shown in Figures S4 and S5.

For the speed of succession, aboveground biomass and maximum height increased fastest in Ghana, at intermediate rates in Mexico, and more slowly in Australia, whereas diversity attributes increased equally fast in Ghana and Mexico but more slowly in Australia (Table 1). Functional composition and biotic interactions changed little during the first 5 years (Table 1), except in Mexico. In both forest types, the percentage of resprouting individuals declined as resprouting is especially high just after land abandonment, whereas afterwards there is continuous regeneration from seed, leading to an increased abundance of seed‐originated individuals (Figures S2i and S3i). In the Mexican dry forest, the percentages of potential nitrogen fixers and arbuscular mycorrhizal–associated species increased, whereas community leaf mass per area and the percentage of biotically dispersed species decreased. In contrast, in the Mexican wet forest, leaf mass per area and biotically dispersed species increased over time (Figure S3).

### 3.3 Dry and Wet Forests Mainly Differ in the Start of Functional Composition and Biotic Interactions

Dry and wet forests differed significantly in the starting values of six attributes and in the speed of three attributes (Figures S4 and S5, Table 1). Wet forests had higher starting values of aboveground biomass, maximum tree height, and the percentage of biotically dispersed species and arbuscular mycorrhizal‐associated species, whereas dry forests had higher community wood density and a greater percentage of nitrogen‐fixers. During the first 5 years of succession, wet forests showed faster increases in maximum tree height, whereas dry forests showed faster increases in the percentage of late‐successional species and faster declines in the percentage of resprouting individuals (Table 1).

## 4 Discussion

We evaluated the start and speed of multidimensional recovery in young secondary forests across six tropical landscapes. We found that (1) successional trajectories showed large variation in forest attributes both across and within the six landscapes, (2) the start and speed of multidimensional succession varied more strongly among countries than between forest types, (3) the start and speed of succession in structure and diversity increased with decreasing land use intensity of the countries, and (4) dry and wet forests differed mainly in the starting values of functional composition and biotic interactions. Below, we discuss the mechanisms underlying these patterns and their implications for multifunctional tropical forest restoration.

### 4.1 Large Variation in the Start and Speed of Succession Across and Within Landscapes

During the first 5 years of succession, structural and diversity attributes increased steadily (Figure 2a–f, cf. Poorter, Craven, et al. 2021), driven by the continuous recruitment of new species and cumulative individual growth. By contrast, functional composition and biotic interactions showed little change, likely due to limited turnover of dominant species during these first 5 years (Finegan 1996; Gómez‐Pompa and Vázquez‐Yanes 1981) and due to the functional similarity among pioneer species (van der Sande et al. 2024). These patterns reveal a partially deterministic component in early succession, consistent with classic successional theories (Clements 1916; Odum 1969): recovery in forest structure and diversity followed similar trajectories across landscapes towards more structurally complex and species‐rich forest stands.

At the same time, substantial variation in both the start and speed of succession occurred among and within landscapes, possibly due to multiple processes operating at different spatial scales. Cross‐site variation (i.e., the *R* ^2^ of the fixed effects) accounted on average for 54% of the total variation in the 12 forest attributes (range: 18%–78%). This reflects substantial differences across countries that differ in overall land‐use history and intensity, as well as between forest types that influence species pools and the length and conditions of the growing season (Table S1). Within‐site variation (i.e., the *R* ^2^ of random plot effects) additionally accounted for an average of 39% of the total variation (range: 19%–70%), indicating differences in successional trajectories among plots within each landscape. This within‐site variation can be attributed to fine‐scale differences such as landscape context (e.g., surrounding forest cover and distance to the nearest forest) that determines the abundance and composition of seed plants and their dispersal vectors in the landscape (Gleason 1926; Pérez‐Cárdenas et al. 2021; Pickett et al. 1987; van Breugel et al. 2025), soil physical and chemical properties (e.g., bulk density and nutrient availability) that influence plant establishment and growth (Lu et al. 2002; Moran et al. 2000), and plot‐level variation in previous land use intensity that shapes biological legacies (Fernandes Neto et al. 2019; Martínez‐Ramos et al. 2016; Zermeño‐Hernández et al. 2016). In sum, succession exhibits predictable increases in structural and diversity attributes during the first 5 years of succession, but the start and speed of successional trajectories differ largely both across and within landscapes.

### 4.2 Countries Differ Strongly in the Start and Speed of Succession of Structure and Diversity

The start and speed of succession in structural and diversity attributes differed strongly amongst countries, with the highest values in Ghana, intermediate values in Mexico, and the lowest in Australia (Figure 2, Table 1). These differences are closely linked to variation in land‐use history and intensity (Table S1). In Mexico and Ghana, steep terrain and high operational costs limit the use of mechanized clearing. As a result, landowners clear their fallow and fields manually with a machete, which leaves behind stumps capable of resprouting (Table S1). Resprouting individuals accounted for 48%–84% of all individuals at the start of succession (Table 1), and these resprouts may contribute to greater maximum tree height and structural heterogeneity in both Mexico and Ghana (Figure 2b,c), as well as high species richness and diversity in Ghana (Figure 2d,e). During the first 5 years of succession, these resprouting individuals, together with seedlings already established during active land use, accelerate structural and diversity recovery (Kammesheidt 1998; Martínez‐Ramos et al. 2021), and thus the recovery of forest multifunctionality (Figure 1). Such high abundances of woody biological legacies (i.e., resprouting individuals and seedlings), particularly in Ghana (Table S1), together with the lower DBH threshold used in this study, help explain the faster recovery of aboveground biomass observed here (wet forest: 72.0 ton ha^−1^ and dry forest: 43.5 ton ha^−1^ after 5 years of succession) compared with average recovery rates of 30.5 ton ha^−1^ reported in Neotropical (Poorter et al. 2016) and 33.0 ton ha^−1^ reported in pantropical studies (Cook‐Patton et al. 2020).

In Australia, pastures have typically been used for much longer periods (over 50 years) and managed more intensively through mechanical clearing (Table S1) (Teitzel 1992; Walker and Weston 1990). As a result, fewer resprouting stumps and seedlings remain at the moment of land abandonment, leading to low observed starting values of structure and diversity (Table S1). The dominance of tall non‐native grasses and ferns at land abandonment (Table S1) further inhibits tree establishment, slowing early recovery and, in some cases, temporarily arresting succession (Catterall 2016; Goosem et al. 2016; Fernandes Neto et al. 2019; Sloan et al. 2016).

Although we used country as a proxy for differences in previous land use duration and intensity (Table S1), our results clearly show that such land‐use legacies have strong effects on both the start and speed of early succession. Future studies should therefore quantify the four main components of land use (type, intensity, extent, and duration) in a standardized way across landscapes (cf. Hordijk, Poorter, Meave, et al. 2024; Martínez‐Ramos et al. 2016; Zermeño‐Hernández et al. 2015) to disentangle their independent and interactive effects on multidimensional forest recovery.

### 4.3 Dry and Wet Forests Differ Strongly in Ecology but Modestly in Successional Pathways

Dry and wet forests represent the two main forest types in the lowland tropics and differ strikingly in their structure, species composition, and functioning (Hall and Swaine 2013; Sanchez‐Azofeifa et al. 2013). Yet, despite these ecological contrasts, their pathways during the first 5 years of succession differed only modestly. Dry and wet forests differed significantly in the starting values of half of the evaluated attributes and in the speed of one quarter (Table 1).

At the start of succession, wet forests showed higher aboveground biomass and maximum tree height (Figure 2a,b), likely due to the presence of taller remnant trees from mature forests (Liu et al. 2025) and/or faster growth of individuals that established during the agricultural phase. Wet forests also showed greater evidence of biotic interactions, with higher percentages of biotically dispersed individuals (Figure 2j), consistent with more pronounced plant–animal co‐evolution in climatically stable ecosystems (Hawes et al. 2020), and a higher percentage of individuals forming arbuscular mycorrhizal associations (Figure 2l), which enhance phosphorus acquisition in highly weathered tropical soils (Vitousek et al. 2010). In contrast, dry forests had higher community wood density and a greater percentage of potential nitrogen fixers (Figure 2g,k), reflecting stronger environmental filtering under hot and dry conditions, in line with previous studies in Neotropical secondary forests (Gei et al. 2018; Poorter et al. 2019). Nitrogen‐fixing species enhance foliar nitrogen and Rubisco concentrations, improving photosynthetic water‐use efficiency (Adams et al. 2016), whereas hardwood species reinforce xylem vessels to reduce the risk of mechanical failure during cavitation (Hacke et al. 2006; Markesteijn et al. 2011). Although dry forest species often exhibit high resprouting capacity in response to drought and fire (Nano and Clarke 2011; Zeppel et al. 2015), we found no significant differences in the percentage of resprouting individuals between forest types (Figure 2i). This may reflect longer land‐use histories and more frequent use of heavy machinery in dry sites in Mexico and Ghana than in wet sites in these countries, which likely reduces the abundance of resprouting stumps and roots and thereby masks potential climatic effects on resprouting.

Differences in the speed of succession were limited (Table 1). Wet forests showed faster increases in maximum tree height, consistent with previous studies, likely because stronger light competition and weaker hydraulic constraints facilitate rapid height growth in these environments (Lang et al. 2023; Lebrija‐Trejos, Meave, et al. 2010). In contrast, the recovery of late‐successional species (i.e., compositional recovery) proceeded more rapidly in dry forests (Figure 2f), possibly due to a relatively small species pool with fewer pioneer species, the strong resprouting capacity of many late‐successional species, and weaker habitat specialization between early‐ and late‐successional species in dry forests (Lebrija‐Trejos et al. 2008; Letcher et al. 2015). Only in Mexico, we observed additional differences in successional trajectories: community leaf mass per area and the percentage of biotically dispersed species decreased significantly in dry forests but increased in wet forests (Figure S3h,j), possibly because these were the driest and wettest sites among the six landscapes and therefore may impose the strongest contrasting environmental filters (Table S1).

In sum, despite strong climatic and ecological contrasts between dry and wet forests, we found surprisingly few differences in both the start and speed of succession during the first 5 years, especially when compared with the pronounced cross‐country differences linked to land‐use history. These findings suggest that succession is the result of a socio‐ecological system (Balvanera et al. 2021; Poorter et al. 2023), and that early successional trajectories are more affected by the social component (i.e., human land‐use practices that modify landscapes and shape biological legacies) than by the ecological component (i.e., forest types). This underscores that (1) socio‐ecological systems must be integrated into successional studies and restoration strategies to better understand and predict forest recovery (Jakovac et al. 2021) and (2) ongoing land‐use change and intensification pose increasing threats to future tropical forest succession and restoration.

### 4.4 Implications for Restoration

The start and speed of secondary tropical forest succession vary widely across landscapes, indicating the need for a portfolio of restoration strategies tailored to local site conditions. In landscapes with short‐duration and non‐mechanized land use, natural regeneration provides a low‐cost, scalable, nature‐based solution to contribute to international commitments such as climate mitigation under the 2015 Paris Agreement (The Paris Agreement | UNFCCC), biodiversity conservation under the 2022 Kunming–Montreal Global Biodiversity Framework (Kunming–Montreal Global Biodiversity Framework), and large‐scale ecosystem restoration under the 2020–2030 UN Decade on Ecosystem Restoration (UN Decade on Restoration). In contrast, in landscapes with intensive and prolonged land‐use histories such as those in Australia, succession can be slow or even arrested due to the dominance of non‐native grasses or ferns and the depletion of resprouting stumps and seedling banks. In such contexts, assisted natural regeneration is recommended, for example, by removing competing herbaceous species with enrichment planting to accelerate tree establishment (Chazdon et al. 2025; Chazdon and Guariguata 2016) or implementing biodiverse tree plantings (Goosem and Tucker 2013; Shoo et al. 2016). Such interventions help overcome barriers to multidimensional recovery.

Where active tree planting is required, species selection should be guided by functional traits suited to local environmental conditions. In dry forests, priority should be given to species with high wood density and nitrogen‐fixing ability, as these traits confer tolerance to drought and heat stress. In wet forests, planting biotically dispersed species can enhance habitat quality and food resources for frugivores, thereby facilitating seed input from surrounding forests and accelerating succession (Elliott et al. 2023). In addition, incorporating species that form arbuscular mycorrhizal associations can facilitate nutrient cycling and promote soil restoration in highly weathered tropical soils. Additionally, species that provide social and economic benefits to local people should be selected (Meli et al. 2014; Prieto‐Rodao et al. 2023).

Together, these insights emphasize that restoration strategies should be tailored to local site conditions and community needs by integrating ecological and social dimensions. Embedding socio‐ecological systems thinking into restoration planning can thus enhance the effectiveness and long‐term success of multifunctional forest restoration in human‐modified tropical landscapes.

## 5 Conclusions

We advanced successional theory in three ways. First, successional trajectories showed large variation in forest attributes both across and within the six landscapes, indicating that succession is shaped by socio‐ecological systems and by local variations such as landscape context, land‐use intensity, and priority effects. Second, the start and speed of succession increased with decreasing previous land‐use intensity, suggesting that biological legacies can kickstart early succession. Third, while forest structure and diversity increased consistently over time, reflecting a deterministic component of succession, functional composition and biotic interactions varied more strongly with forest type, highlighting the role of environmental filtering. Together, these findings highlight the importance of integrating early successional dynamics, socio‐ecological context, and multiple dimensions of forest recovery to better understand and predict forest succession and to scale restoration efforts in human‐modified tropical landscapes.

## Author Contributions

**Frans Bongers:** methodology, investigation, writing – review and editing. **Lucy Amissah:** methodology, investigation, supervision, writing – review and editing. **Tomonari Matsuo:** conceptualization, methodology, data curation, investigation, formal analysis, visualization, writing – original draft, writing – review and editing. **Lourens Poorter:** conceptualization, methodology, investigation, data curation, supervision, project administration, funding acquisition, writing – review and editing, writing – original draft. **Iris Hordijk:** methodology, investigation, writing – review and editing, visualization, supervision. **Laura Marteijn:** methodology, investigation, writing – review and editing. **Masha T. van der Sande:** methodology, investigation, supervision, writing – review and editing, funding acquisition. **Jazz Kok:** investigation, methodology, writing – review and editing. **Susan G. W. Laurance:** methodology, investigation, writing – review and editing. **Jorge A. Meave:** methodology, investigation, funding acquisition, writing – review and editing. **Miguel Martínez‐Ramos:** writing – review and editing, methodology, investigation, funding acquisition. **Luis Octavio Zavala:** methodology, investigation, writing – review and editing.

## Conflicts of Interest

The authors declare no conflicts of interest.

## Supporting information


**Figure S1:** gcb70874‐sup‐0001‐Supinfo.docx.
**Figure S2:** gcb70874‐sup‐0001‐Supinfo.docx.
**Figure S3:** gcb70874‐sup‐0001‐Supinfo.docx.
**Figure S4:** gcb70874‐sup‐0001‐Supinfo.docx.
**Figure S5:** gcb70874‐sup‐0001‐Supinfo.docx.
**Table S1:** gcb70874‐sup‐0001‐Supinfo.docx.

## Data Availability

Raw data on 12 forest attributes and calculated ecosystem multifunctionality are stored in Data Archiving and Networked Services (https://doi.org/10.17026/LS/P3I4MN).
