# Supplementary material for: Multidimensional Recovery of Young Secondary Forests in Human‐Modified Tropical Landscapes
Source: Glob Chang Biol. 2026 Apr 17;32:e70874. doi: 10.1111/gcb.70874 (PMC13090750; doi:10.1111/gcb.70874)
Supplement: Supplementary file 1 — Figure S1: gcb70874‐sup‐0001‐Supinfo.docx. Figure S2: gcb70874‐sup‐0001‐Supinfo.docx. Figure S3: gcb70874‐sup‐0001‐Supinfo.docx. Figure S4: gcb70874‐sup‐0001‐Supinfo.docx. Figure S5: gcb70874‐sup‐0001‐Supinfo.docx. Table S1: gcb70874‐sup‐0001‐Supinfo.docx. [file GCB-32-e70874-s001.docx]

**Multidimensional recovery of young secondary forests in human-modified tropical landscapes**

*Supplementary Information*

**Authors.** Tomonari Matsuo^1*^, Lourens Poorter^1^, Lucy Amissah^2,3^, Susan G. W. Laurance^4^, Miguel Martínez-Ramos^5^, Jorge A. Meave^6^, Frans Bongers^1^, Masha T. van der Sande^1^, Jazz Kok^1^, Laura Marteijn^1^, Luis Octavio Zavala^6^, Iris Hordijk^1^

^1^Forest Ecology and Forest Management Group, Wageningen University & Research, P.O. Box 47, 6700 AA Wageningen, The Netherlands.

^2^Council for Scientific and Industrial Research-Forestry Research Institute of Ghana, Kumasi, Ghana

^3^CSIR College of Science and Technology, P.O. Box M 32, Accra

^4^Centre for Tropical Environmental and Sustainability Science (TESS), James Cook University, Cairns, QLD, Australia

^5^Instituto de Investigaciones en Ecosistemas y Sustentabilidad, Universidad Nacional Autónoma de México, CP 58190, Morelia, Michoacán, México

^6^Departamento de Ecología y Recursos Naturales, Facultad de Ciencias, Universidad Nacional Autónoma de México, Coyoacán, Mexico City 04510, Mexico

^*^Corresponding author: email: [tomonari.matsuo@wur.nl](mailto:tomonari.matsuo@wur.nl)

Table S1. Overview of site characteristics of the six study landscapes. The table summarizes key site characteristics: latitude and longitude; mean annual temperature (MAT, ℃); mean annual precipitation (MAP, mm year^-1^); dry season duration (mean monthly precipitation < 100 mm) (Goosem et al., 2016; Hordijk et al., 2024; Matsuo et al., 2025); Human Development Index (HDI: composite index of life expectancy, education, and income per capita) (Baumann, 2021); position along the forest transition curve (FT curve: stage of forest cover change along the forest transition trajectory, from net deforestation to forest recovery) (de Jong et al., 2025); primary land-use (LU) type (pasture vs. crop); land-use duration (average years of previous land use across plots estimated from interviews with landowners and complemented by historical aerial photography); land-use intensity (percentage of plots where mechanized agriculture was used for field preparation); leaf area index (LAI, m^2^ m^-2^) of grasses and ferns between 0 and 2 m height along four 25-m transects at time of land abandonment (stand age=0); number of resprouting individuals (DBH ≥ 1 cm) per plot (625 m^2^) at land abandonment; number of seedlings (DBH<1cm, height>30cm) per plot along four 1 m × 25 m transects (100 m^2^) at land abandonment; most abundant woody plant family based on all the permanent plot data (Dominant fam); number of plots; and monitoring period (years). Different letters in the table indicate significant differences among landscapes based on generalized linear models with a negative binomial distribution (seedlings), and linear models with square-root–transformed response variables (resprouting individuals and leaf area index of grasses and ferns).

| **Site** | **Australia-Dry** | **Australia-Wet** | **Mexico-Dry** | **Mexico-Wet** | **Ghana-Dry** | **Ghana-Wet** |
| --- | --- | --- | --- | --- | --- | --- |
| **Latitude** | 17°19′S | 17°23′S | 16°39′N | 16°05′ N | 7°08’N | 5°09’N |
| **Longitude** | 145°31′E | 145°35′E | 95°00′ W | 91°00′ W | 1°45’ W | 1°58’ W |
| **MAT** | 20.8 | 20.8 | 27.7 | 24.4 | 25.6 | 26.0 |
| **MAP** | 1584 | 2077 | 900 | 3000 | 1290 | 1808 |
| **Dry season** | Jul-Oct | Jul-Oct | Nov-Apr | Feb-Apr | Nov-Feb | Dec-Feb |
| **HDI** | Very high | Very high | High | High | Medium | Medium |
| **FT curve** | Post | Post | Late | Late | Early | Early |
| **LU type** | Pasture | Pasture | Crop / Pasture | Pasture | Crop | Crop |
| **LU duration** | Long (>50 years) | Long (>50 years) | Short (21.8) | Short (19.2) | Short (15.4) | Short (10.7) |
| **LU intensity** | 68.4 | 76.5 | 50 | 5.0 | 0 | 0 |
| **Grass & fern** | 2.9 (0.0096-7.5) ^b^ | 4.0 (0.82-9.1) ^b^ | 0.36 (0-2.0) ^a^ | 0.71 (0.27-1.3) ^a^ | 0.63 (0.087-1.4) ^a^ | 0.81 (0.18-1.4) ^a^ |
| **Resprouts** | 1 (0-10) ^a^ | 1 (0-16) ^a^ | 22 (0-90) ^bc^ | 9 (0-30) ^b^ | 57 (4-128) ^d^ | 44 (3-146) ^cd^ |
| **Seedlings** | 2 (0-15) ^a^ | 3 (0-30) ^a^ | 24 (0-138) ^b^ | 17 (0-115) ^b^ | 46 (21-76) ^bc^ | 295 (126-562) ^c^ |
| **# Plots** | 21 | 21 | 21 | 20 | 20 | 19 |
| **Monitoring** | 2021-2025 | 2022-2025 | 2020-2024 | 2020-2024 | 2021-2024 | 2021-2024 |


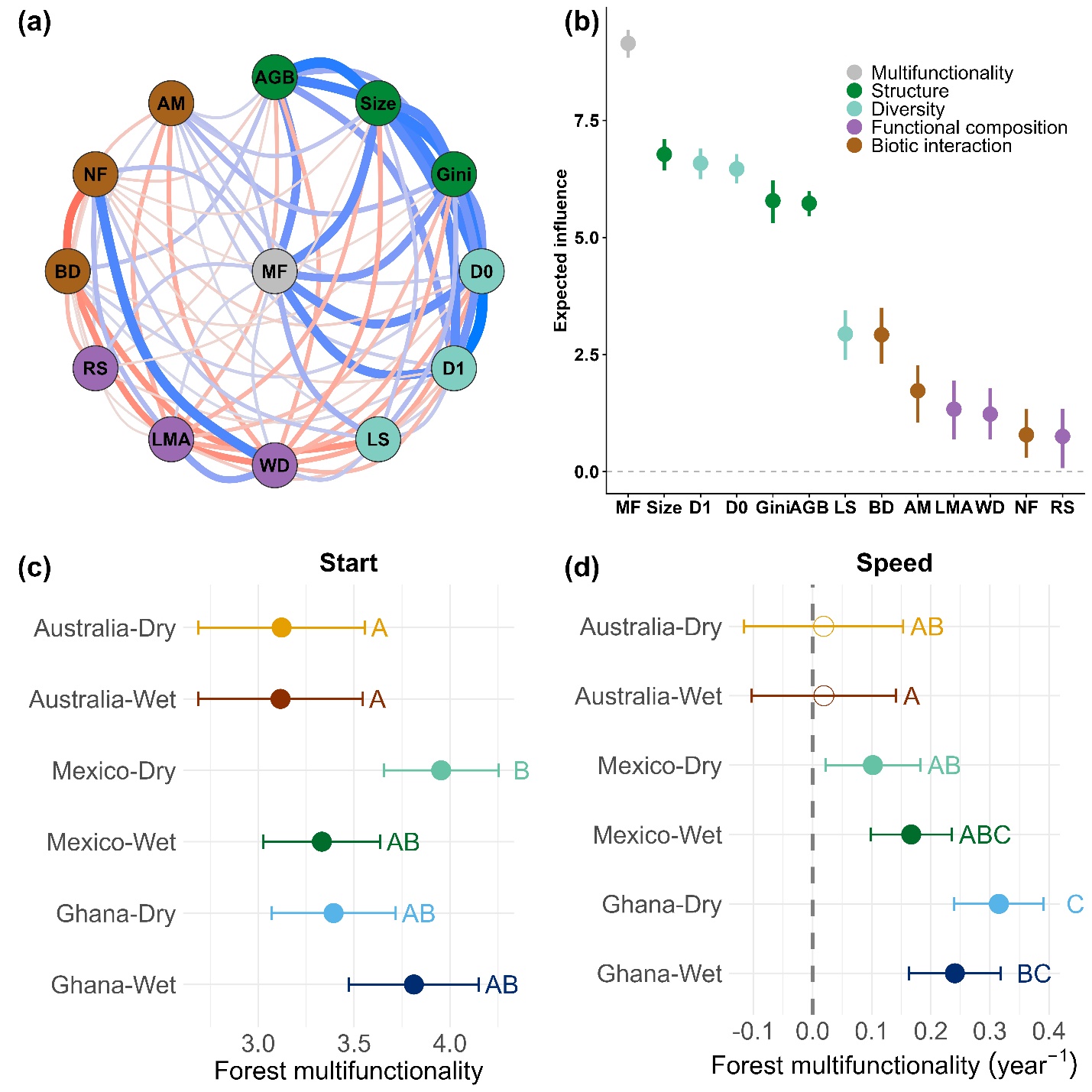


**Figure S1. Forest multifunctionality across six landscapes.** (a) Correlation network among 12 forest attributes and overall multifunctionality, based on all plot-census combinations. Line color and thickness indicate the direction and strength of correlations (blue = positive, red = negative); darker and thicker lines represent stronger relationships. Lines are shown only for significant pairwise correlations (i.e., with a 95% confidence interval that does not overlap with zero). (b) Expected influence of each attribute within the correlation network among 12 forest attributes and overall multifunctionality. The expected influence quantifies the relative importance of each attribute within the network and was calculated as the sum of its partial correlations with all other attributes. Edge weights and expected influence values were estimated from 10,000 bootstraps of the empirical network, and 95% confidence intervals were derived from the bootstrap distributions. (c, d) Estimated marginal means (± 95% confidence interval) of the (c) start (initial value; intercept) and (d) speed (annual rate of change; slope) of forest multifunctionality across six sites/landscapes (country-forest type combinations). A linear mixed-effects model was fitted with multifunctionality as the response variable, country (Ghana, Mexico, Australia), forest type (dry, wet), stand age, and their three-way interactions as fixed effects, while plot was included as a random intercept and slope to account for repeated measurements across censuses. Letters denote significant post-hoc differences among landscapes. Closed circles indicate significant (c) intercepts or (d) slopes, while open circles indicate non-significant values. Attribute abbreviations: multifunctionality (MF), aboveground biomass (AGB; ton ha^-1^), diameter of the third thickest individual (Size; cm), Gini coefficient of tree basal area within each plot (Gini), species richness based on Hill number of order 0 (D_0_; per 625 m^2^), exponentiated Shannon diversity based on Hill number of order 1 (D_1_; per 625 m^2^), percentage of late-succession species (LS; %), community-weighted mean wood density (WD; g cm^-3^), community-weighted mean leaf mass per area (LMA; g m^-2^), percentage of resprouting individuals (RS; %), percentage of biotically dispersed trees (BD; %), percentage of nitrogen-fixing trees (NF; %), and percentage of trees with the capacity to form arbuscular mycorrhizal association (AM; %). For a detailed description of the attributes, see the Materials and Methods section.


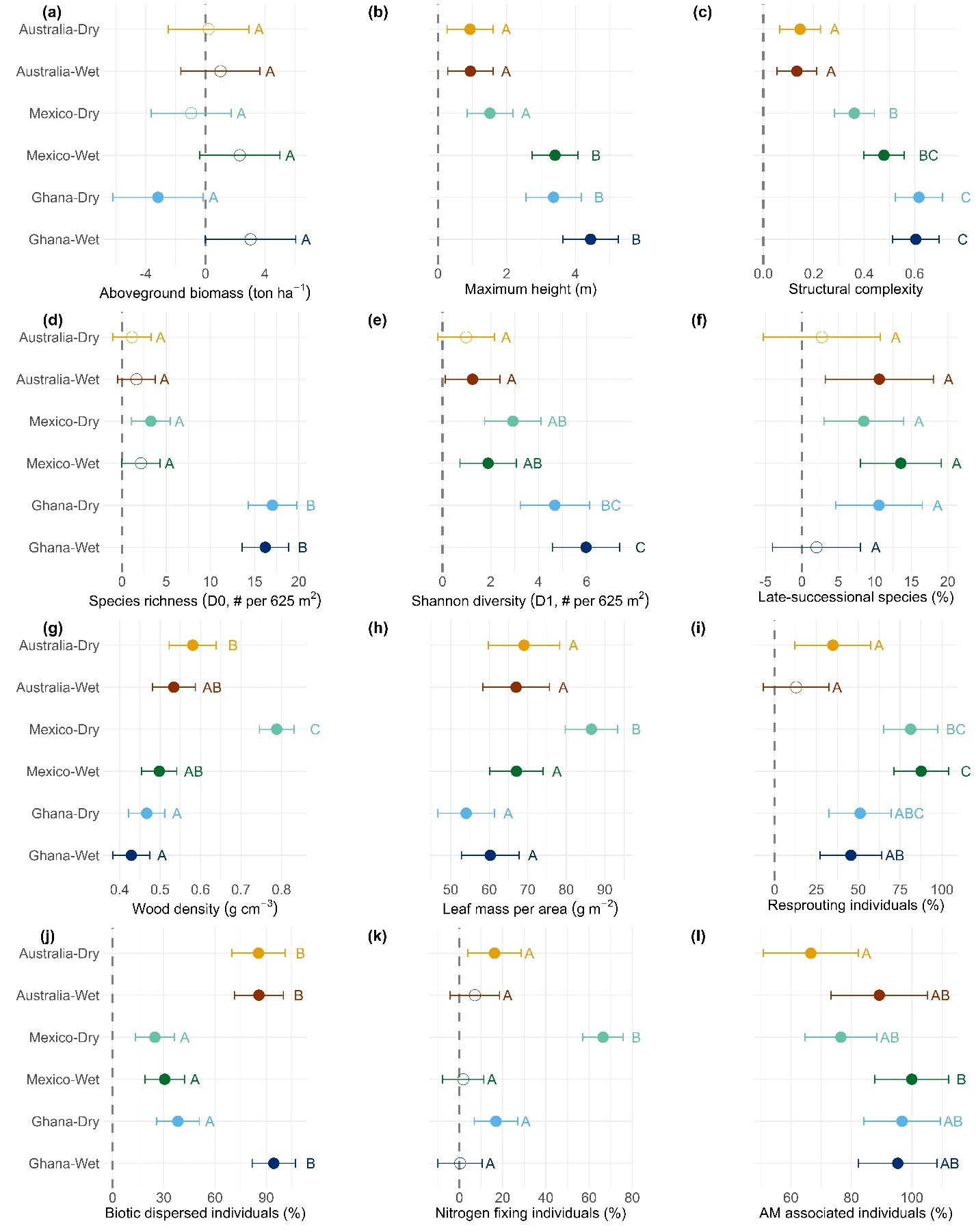


**Figure S2. Intercepts of 12 forest attributes across six landscapes (start of succession, stand age = 0).** Panels show estimated marginal means of intercepts (initial value) with ± 95% confidence intervals from linear mixed models for (a) aboveground biomass, (b) maximum tree height (the height of the third tallest individual), (c) structural complexity based on Gini coefficient of tree basal area within the plot, (d) species richness based on Hill number of order 0, (e) exponentiated Shannon diversity based on Hill number of order 1, (f) percentage of late-successional species, (g) community-weighted mean wood density, (h) community-weighted mean leaf mass per area, (i) percentage of resprouting individuals (RS), (j) percentage of biotically dispersed trees (BD), (k) percentage of nitrogen-fixing trees (NF), and (l) percentage trees with the capacity to form arbuscular mycorrhizal association (AM). For a detailed description of the attributes, see the Materials and Methods section. For each attribute, a linear mixed-effects model was fitted with country (Ghana, Mexico, Australia), forest type (dry, wet), stand age, and their three-way interactions as fixed effects, while plot was included as a random intercept and slope to account for repeated measurements across censuses. Capital letters indicate post-hoc comparisons among six landscapes. Closed circles denote significant intercepts, whereas open circles denote non-significant intercepts.

**
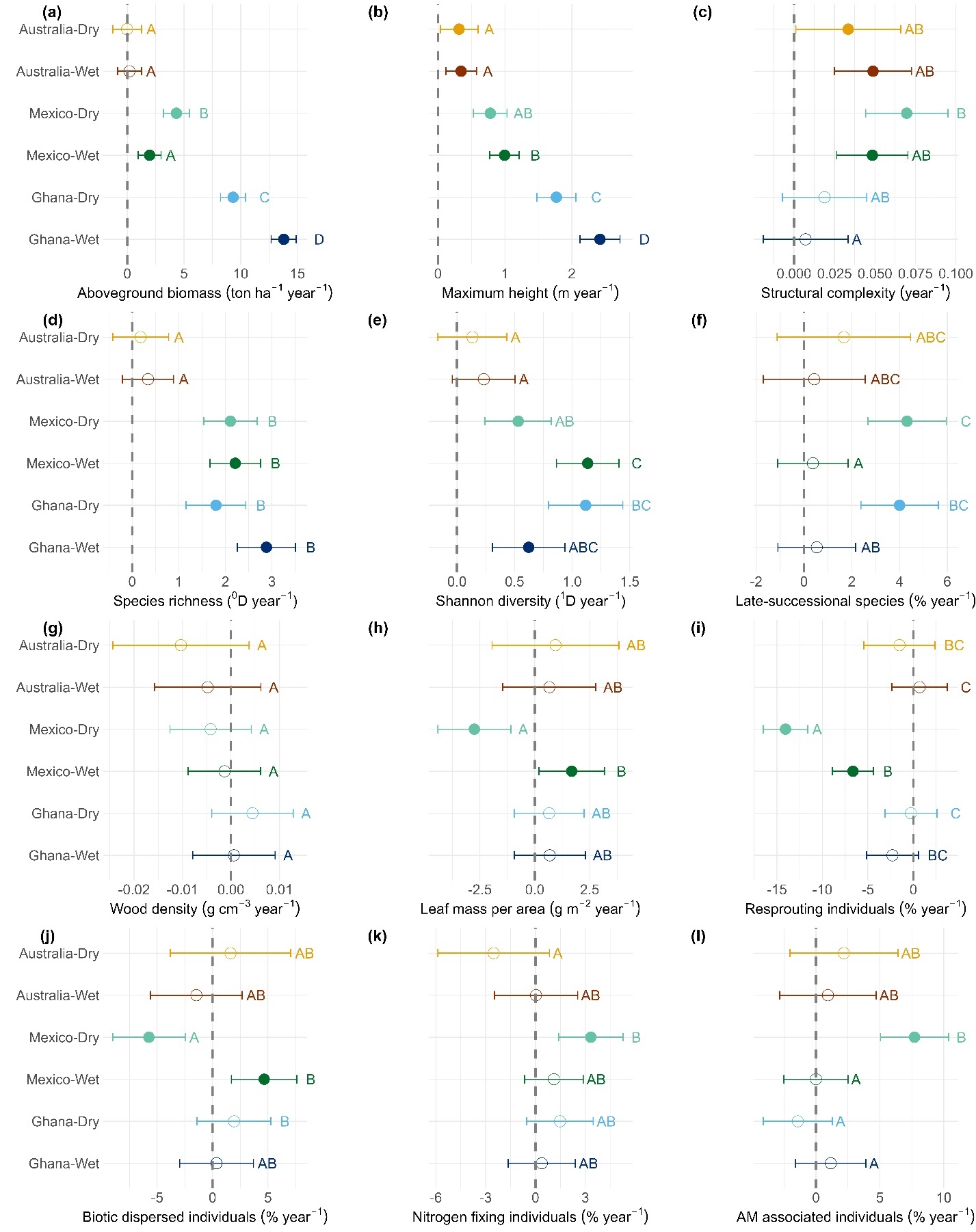
**

**Figure S3. Slopes of 12 forest attributes across six landscapes (speed of succession, per year).** Panels show estimated marginal means of slopes (annual rate of change) with ± 95% confidence interval from linear mixed models for (a) aboveground biomass, (b) maximum tree height (the height of the third tallest individual), (c) structural complexity based on Gini coefficient of tree basal area within the plot, (d) species richness based on Hill number of order 0, (e) exponentiated Shannon diversity based on Hill number of order 1, (f) percentage of late-successional species, (g) community-weighted mean wood density, (h) community-weighted mean leaf mass per area, (i) percentage of resprouting individuals (RS), (j) percentage of biotically dispersed trees (BD), (k) percentage of nitrogen-fixing trees (NF), and (l) percentage trees with the capacity to form arbuscular mycorrhizal association (AM). For a detailed description of the attributes, see the Materials and Methods section. For each attribute, a linear mixed-effects model was fitted with country (Ghana, Mexico, Australia), forest type (dry, wet), stand age, and their three-way interactions as fixed effects, while plot was included as a random intercept and slope to account for repeated measurements across censuses. Capital letters indicate post-hoc comparisons among six landscapes. Closed circles denote significant slopes, whereas open circles denote non-significant slopes.


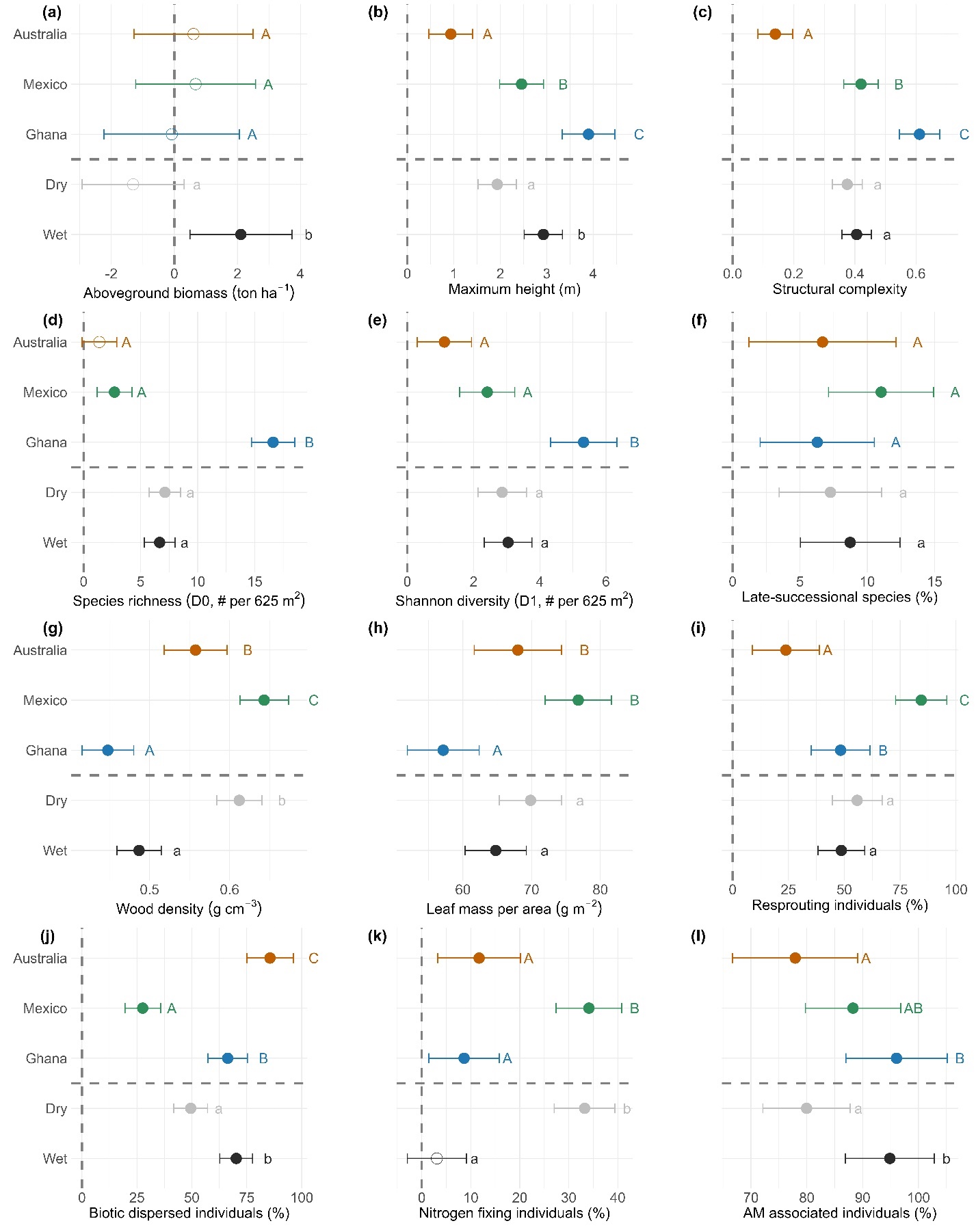


**Figure S4. Intercepts of 12 forest attributes across countries and climates (start of succession, stand age = 0).** Panels show estimated marginal means of intercepts (initial values) with ± 95% confidence interval from linear mixed models for (a) aboveground biomass, (b) maximum tree height (the height of the third tallest individual), (c) structural complexity based on Gini coefficient of tree basal area within the plot, (d) species richness based on Hill number of order 0, (e) exponentiated Shannon diversity based on Hill number of order 1, (f) percentage of late-successional species, (g) community-weighted mean wood density, (h) community-weighted mean leaf mass per area, (i) percentage of resprouting individuals (RS), (j) percentage of biotically dispersed trees (BD), (k) percentage of nitrogen-fixing trees (NF), and (l) percentage trees with the capacity to form arbuscular mycorrhizal association (AM). For a detailed description of the attributes, see the Materials and Methods section. For each attribute, a linear mixed-effects model was fitted with country (Ghana, Mexico, Australia), forest type (dry, wet), stand age, and their three-way interactions as fixed effects, while plot was included as a random intercept and slope to account for repeated measurements across censuses. Capital letters indicate post-hoc comparisons among countries, and lowercase letters indicate post-hoc comparisons between dry and wet forests. Closed circles denote significant intercepts, whereas open circles denote non-significant intercepts.


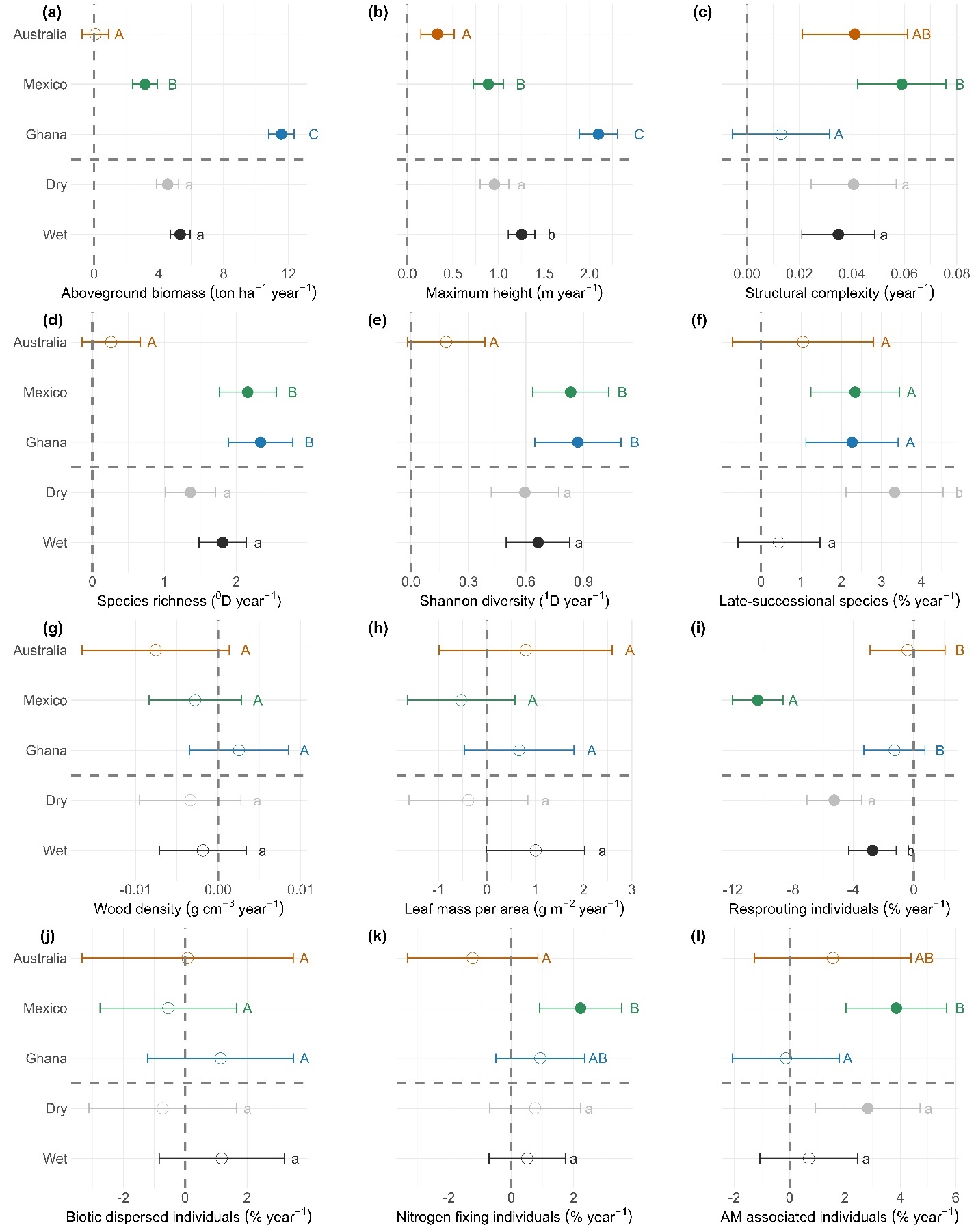


**Figure S5. Slopes of 12 forest attributes across countries and climates (speed of succession, per year).** Panels show estimated marginal means of slopes (annual rate of change) with ±95% confidence interval from linear mixed models for (a) aboveground biomass, (b) maximum tree height (the height of the third tallest individual), (c) structural complexity based on Gini coefficient of tree basal area within the plot, (d) species richness based on Hill number of order 0, (e) exponentiated Shannon diversity based on Hill number of order 1, (f) percentage of late-successional species, (g) community-weighted mean wood density, (h) community-weighted mean leaf mass per area, (i) percentage of resprouting individuals (RS), (j) percentage of biotically dispersed trees (BD), (k) percentage of nitrogen-fixing trees (NF), and (l) percentage trees with the capacity to form arbuscular mycorrhizal association (AM). For a detailed description of the attributes, see the Materials and Methods section. For each attribute, a linear mixed-effects model was fitted with country (Ghana, Mexico, Australia), forest type (dry, wet), stand age, and their three-way interactions as fixed effects, while plot was included as a random intercept and slope to account for repeated measurements across censuses. Capital letters indicate post-hoc comparisons among countries, and lowercase letters indicate post-hoc comparisons between dry and wet forests. Closed circles denote significant slopes, whereas open circles denote non-significant slopes.

**References**

Baumann, F. (2021). The Next Frontier—Human Development and the Anthropocene: UNDP Human Development Report 2020. *Environment*, *63*(3), 34–40. https://doi.org/10.1080/00139157.2021.1898908

de Jong, J., Poorter, L., de Jong, W., Bongers, F., Lohbeck, M., Veenendaal, E., Meave, J. A., Jakovac, C. C., Brancalion, P. H. S., Amissah, L., Martínez-Ramos, M., Bartholomeus, H., Laurance, S. G. W., Brown, W. H., & Decuyper, M. (2025). Dissecting forest transition: Contribution of mature forests, second-growth forests and tree plantations to tree cover dynamics in the tropics. *Land Use Policy*, *153*. https://doi.org/10.1016/j.landusepol.2025.107545

Goosem, M., Paz, C., Fensham, R., Preece, N., Goosem, S., & Laurance, S. G. W. (2016). Forest age and isolation affect the rate of recovery of plant species diversity and community composition in secondary rain forests in tropical Australia. *Journal of Vegetation Science*, *27*(3), 504–514. https://doi.org/10.1111/jvs.12376

Hordijk, I., Poorter, L., Meave, J. A., Bongers, F., van der Sande, M. T., López Mendoza, R. D., Jamangapé Romero, P., de Jong, J., & Martínez-Ramos, M. (2024). Land use history and landscape forest cover determine tropical forest recovery. *Journal of Applied Ecology*. https://doi.org/10.1111/1365-2664.14754

Matsuo, T., Poorter, L., van der Sande, M. T., Mohammed Abdul, S., Koyiba, D. W., Opoku, J., de Wit, B., Kuzee, T., & Amissah, L. (2025). Drivers of biomass stocks and productivity of tropical secondary forests. *Ecology*, *106*(1), e4488. https://doi.org/10.1002/ecy.4488
